# Effect of hepatic sympathetic nerve removal on energy metabolism in an animal model of cognitive impairment and its relationship to *Glut2* expression

**DOI:** 10.1515/biol-2020-0033

**Published:** 2020-06-05

**Authors:** Riming Wei, Xiuhong Zhuge, Pengpeng Yue, Manjun Liu, Lin Zhu, Jianxiang Liu, Chunbo Xia

**Affiliations:** College of Biotechnology, Guilin Medical University, Guilin, Guangxi, 541004, China; Affiliated Hospital of Guilin Medical University, Guilin, Guangxi, 541004, China; Department of Human Anatomy of Basic Medical College, Guilin Medical University, Guilin, Guangxi, 541004, China

**Keywords:** energy metabolism, rats with cognitive impairment, hepatic sympathetic nerve, glucose transporter 2 (*Glut2*)

## Abstract

The aims of this study were to investigate the effect of hepatic sympathetic nerve removal on glucose and lipid metabolism in rats with cognitive impairment and to evaluate the relationship between these effects and liver *Glut2* expression. Hippocampal injection of Aβ_1–42_ was used to induce cognitive impairment. Impaired rats were divided into experimental, sham, and control groups. The experimental group was injected with 6-hydroxydopamine to remove the sympathetic nerve. At 4 weeks post injection, body weight, food and water intake, blood sugar, and blood lipids were measured, and periodic acid-Schiff (PAS) staining was used to assess the liver glycogen content. Liver *Glut2* mRNA and protein were also detected. The experimental group showed reduced body weight, food intake, and blood glucose levels and elevated insulin levels compared with the control group. PAS staining showed higher glycogen contents in the experimental group than in controls. The expression levels of *Glut2* mRNA and protein in the experimental group were significantly lower than in the controls. Metabolism was significantly impacted in rats with cognitive impairment following removal of the hepatic sympathetic nerve. Disruption to *Glut2* liver expression via sympathetic nerve disruption represents a possible underlying mechanism.

## 1 Introduction

The nervous system detects internal and external fluctuations and rapidly triggers the appropriate physiological response. The central nervous system (CNS) regulates energy metabolism [1,2] and receives metabolic status signals, including leptin, insulin, growth hormone-releasing peptide, thyroid hormone, and gonadal hormone, from the periphery. These signals are delivered via the afferent nerve to the CNS, where they are integrated and analyzed and then directed to the efferent nerve to regulate different physiological processes, including energy balance, energy consumption, and peripheral metabolism [3,4,5]. The autonomic nervous system (ANS) includes sympathetic and parasympathetic nerves, which jointly innervate surrounding metabolic tissues, including brown and white adipose tissues, liver, pancreas, and skeletal muscle. Sympathetic and parasympathetic nerves are antagonistic to each other, an effect that plays an important role in response to peripheral signals. Changes in CNS functioning, especially hypothalamus, not only affect liver energy metabolism but also affect the function of other metabolic organs [6]. In the study of the effects of the ANS on glucose and lipid metabolism, López-Soldado et al. have shown that the vagus liver branch is involved in regulating food intake and glucose homeostasis in mice [7]. Chronic electrical stimulation of the vagus nerve branch of bilateral minipigs can significantly improve diet-induced obesity insulin sensitivity [8]. The activation of extracellularly regulated kinase in mouse liver can cause pancreatic β-cell proliferation under the action of the nervous system, thereby reducing blood glucose levels [9]. In addition, the vagus nerve has a complex connection with the visceral central hippocampus [10]. Epidemiological studies indicate that disrupted glucose and lipid metabolism frequently occurs in Alzheimer’s disease (AD) [11]. Similarly, animal studies have shown elevated glucose and lipid levels in rats with cognitive impairment [12]. The liver is the primary site of glucose uptake, storage, and metabolism. Therefore, we postulate that sympathetic nervous system impairment leads to disrupted liver function. To further clarify this relationship, we removed the hepatic sympathetic nerve from cognitively impaired rats and examined the effects on body weight, food and water intake, blood glucose, blood lipids, insulin, liver glycogen content, and liver tissue *Glut2* expression levels.

## 2 Materials and methods

### 2.1 Instruments and laboratory reagents

An automatic biochemical analyzer (Erba XL-600), refrigerated centrifuge (Cene H1650), fluorescence quantitative PCR instrument (Bio-Rad MYIQ2 and CFX96), light microscope (Olympus), gel imaging system (Bio-Rad), Rheodyne valve (Shanghai Gauge), Aβ_1–42_ (Sigma, A9810), 6-hydroxydopamine (6-OHDA; Sigma, H4381), TRIzol reagent (Invitrogen, 15596026), blood glucose and lipid assay kits (Ulite Biotechnology Co. U82980030), fluorescence quantitative PCR kit (Takara Bio, RR037A), anti-GLUT2 antibody (Beijing Bioss Antibodies Co. bs-0351R), antibody for β-actin (Abcam Co. 8227), BCA protein quantification kit (Beyotime Biotechnology, P0012S), and glycogen PAS staining kit (Solarbio Life Sciences, G1280) were used.

The following primer sequences were used: *Glut2*, 5′-TCTGTGCTGCTTGTGGAG-3′ and 5′-AGAGGGCGATGATGAAAT-3′; β-actin, 5′-CCCATCTATGAGGGTTACGC-3′ and 5′-CCCATCTATGAGGGTTACGC-3′. All primers were purchased from Proteintech Co.

### 2.2 Animal treatment and experimental groups

Male SD rats of weight 300 ± 10 g were obtained from the Guilin Medical University SPF-grade experimental animal center (certificate #SCXK GUI 2007-0001). To induce cognitive impairment, rats were anesthetized with 0.3% pentobarbital sodium (40 mg/kg) followed by a hippocampal injection of 15 µg of Aβ_1–42_ according to the stereosteric map of the rat brain and using a stereosteric instrument. The injection site was located 3.8 mm behind the anterior fontanelle and 3.3 mm from the right side of the brain.

Cognitive ability assessments were conducted 60 days post injection using a water maze. The time between entering the water and climbing onto the platform, the time spent crossing the platform, and the percentage of time spent in the initial platform quadrant were recorded. Rats with cognitive impairment were randomly assigned to the experimental group (*n* = 15), sham group (*n* = 15), or control group (*n* = 15). The liver sympathetic nerve of rats does not form a neural stem, and it is distributed. The sympathetic nerve can selectively take up 6-OHDA and deplete the inner catecholamines, which causes neuronal degeneration. The method of removing liver sympathetic nerves was outlined by Lin et al. [13]. After abdominal anesthesia, 6-OHDA (1.0 mg/kg) was injected into the mesenteric vein with a scalp needle and delivered to the liver through the portal vein system. Sham-group animals were injected with an equal volume of normal saline, and the control group rats received no injection.

The rats in this experiment were raised in metabolic cages, one rat per cage, and adequate feed and water were provided. Food and water intakes (feed intake = feed added − residual amount; water intake = initial water amount − residual water amount) were measured 3 days before the experiment and during the experiment as indicated below.


**Ethical approval:** The research related to animals’ use has been complied with all the relevant national regulations and institutional policies for the care and use of animals, and has been approved by the Guilin Medical University Ethic Committee (ethical approval number: GLMC201703022).

### 2.3 Tissue collection and measurement of blood glucose, lipid, and insulin levels

Four weeks after the 6-OHDA injection, the rats were fasted for 12 h, weighed, and given abdominal anesthesia (0.3% sodium pentobarbital, 40 mg/kg). After anesthesia, the rats were fixed on the operating table with a midline neck incision to expose the common carotid artery, 10 mL of blood was collected from the common carotid artery, and the serum was isolated by centrifugation. Liver tissue samples were collected and stored at −80°C. Serum insulin levels were quantitated by enzyme-linked immunosorbent assay. An automatic biochemical analyzer was used to measure the triglyceride (TG), total cholesterol (TC), high-density lipoprotein cholesterol (HDL), and low-density lipoprotein cholesterol (LDL) levels. The fasting blood glucose level was measured using a Roche glucometer. All experiments were replicated three times.

### 2.4 PAS method for detection of liver glycogen

Liver tissues were ethanol dehydrated for 2 h, paraffin-embedded, and sectioned (4 µm). Dimethyl benzene dewaxing was followed by tissue rehydration. Next, the sections were placed into an oxidant at room temperature (25–30°C) for 5 min, rinsed, and submerged twice in distilled water. The sections were next kept in Schiff’s colorless magenta solution at room temperature (25–30°C) for 15 min, rinsed in distilled water for 10 min, immersed in hematoxylin for 2 min and then in acidic solution for 2 s, and rinsed in running distilled water for 15 min. Double-distilled water was then used to rinse until tissues appeared blue. Finally, the sections were made transparent by xylene and mounted on slides with neutral gum. Image analysis software was used to calculate the average optical density of PAS-positive argentaffin cells as a representation of the liver glycogen content. Unstained Schiff’s colorless magenta solution was used as the negative control. At least five images randomly selected from one liver tissue were analyzed for the calculation of the optical density of PAS-positive argentaffin cells.

### 2.5 Quantitative PCR analysis of liver *Glut2* mRNA expression

Liver tissues were ground into powder using liquid nitrogen, and total RNA was isolated using the TRIzol reagent. The RNA was precipitated using chloroform and isopropanol and dissolved in RNase-free water. The RNA quantity and quality were determined prior to reverse transcription. *Glut2* mRNA levels were measured by real-time quantitative PCR (qPCR) using the following cycle conditions: 95°C for 10 min, followed by 40 cycles of 95°C for 15 s and 60°C for 15 s. A dissociation curve was generated for all qPCR products by incrementally (0.3°C) increasing the temperature from 60 to 95°C. CT values were calculated by the instrument software, and relative expression was expressed as 2^−ΔΔCT^ values. The RNA quantity and quality of every sample were determined three times.

### 2.6 Detection of liver GLUT2 expression by Western blot

Lysis buffer was added to ground liver tissue samples at a ratio of 100 µL per 20 mg of tissue to isolate total protein. Protein concentrations were determined using the BCA protein quantification kit. Western blot analysis was conducted by SDS-PAGE gel electrophoresis followed by transfer to the polyvinylidene fluoride (PVDF) membrane at 200 mA for 1.5 h. GLUT2 primary antibody (20436-1-AP; Proteintech Group, Inc.) was applied to the membrane followed by overnight incubation at 4°C and then washing. The membrane was next incubated with secondary antibody (074-1506, KPL) at room temperature for 1 h followed by incubation with ECL chemiluminescence substrates and exposed to X-ray film, which was analyzed using the Bio-Rad imaging system. The gray value of the GLUT2 protein was divided by the gray value of the internal reference gene (β-actin) to determine the relative amount of GLUT2 in the sample.

### 2.7 Statistical analysis

SPSS version 17.0 statistical analysis software was used to analyze the experimental results, and the data were expressed as arithmetic mean and standard deviation \left(\bar{x}\pm s\right). One-way analysis of variance was used to detect differences among multiple groups. Dunnett’s statistical method was used to determine the significance, and the significance threshold was set to 0.05 for all tests.

## 3 Results

### 3.1 Blood glucose, lipid, and insulin levels

Four weeks after 6-OHDA injection, the body mass and daily food intake of the experimental group rats were 292 ± 12.33 g and 18.71 ± 10.88 g, respectively, which were significantly lower than those of the sham (356 ± 9.41 g and 26.44 ± 9.73 g, respectively) and control groups (363 ± 13.25 g and 27.52 ± 10.62 g, respectively) (*P* < 0.01). There was no significant difference in water intake among the groups (*P* > 0.05) (Table 1). Significant differences were detected between the fasting blood glucose and insulin levels of the experimental group (6.28 ± 1.86 mmol/L and 35.40 ± 6.84 IU/L, respectively) and the control group (7.53 ± 1.24 mmol/L and 26.45 ± 6.71 IU/L, respectively) (*P* < 0.01). However, there were no significant differences in TG, TC, HDL, or LDL levels (*P* > 0.05). No differences were detected between the sham group and the control group (*P* > 0.05, Table 2).

**Table 1:** Weight, feed intake, and water intake \left(\bar{x}\pm s\right) in rats with cognitive impairment 4 weeks after the removal of the hepatic sympathetic nerve

Group	*n*	Weight (g)	Daily food intake (g/rat)	Daily quantity of drinking water (ml/rat)
Experimental group	15	292 ± 12.33*	18.71 ± 10.88*	31.45 ± 12.49**
Sham group	15	356 ± 9.41**	26.44 ± 9.73**	32.80 ± 9.56**
Control group	15	363 ± 13.25	27.52 ± 10.62	31.39 ± 8.92

Compared to the control group, *P < 0.01, **P > 0.05.

**Table 2:** Blood glucose, blood lipid, and insulin levels \left(\bar{x}\pm s\right) in rats with cognitive impairment 4 weeks after the removal of the hepatic sympathetic nerve

Group	*n*	FPG (mmol/L)	TG (mmol/L)	TC (mmol/L)	LDL (mmol/L)	HDL (mmol/L)	INS (µIU/L)
Experimental group	15	6.28 ± 1.86*	0.81 ± 0.43**	1.63 ± 0.30**	0.66 ± 0.16**	0.78 ± 0.33**	35.40 ± 6.84*
Sham group	15	7.82 ± 1.30**	0.78 ± 0.37**	1.58 ± 0.56**	0.69 ± 0.23**	0.76 ± 0.28**	25.32 ± 7.36**
Control group	15	7.53 ± 1.24	0.82 ± 0.26	1.59 ± 0.29	0.71 ± 0.25	0.80 ± 0.24	26.45 ± 6.71

Compared to the control group, *P < 0.01, **P > 0.05.

### 3.2 Glycogen content in liver tissue

**Figure 1:**
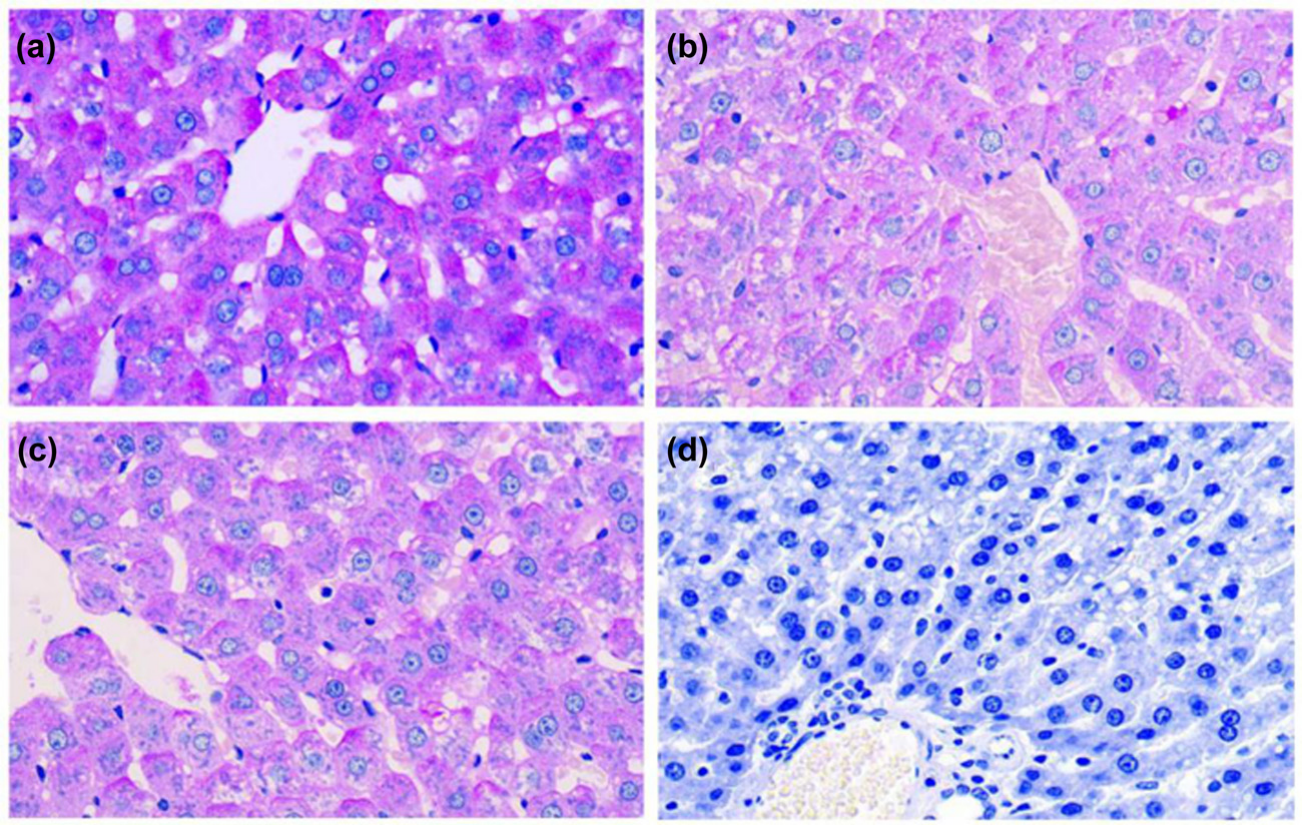
Liver glycogen content in rats with cognitive impairment 4 weeks after liver sympathetic nerve removal (PAS, ×400) (a) experimental group, (b) sham group, (c) control group, and (d) negative control.

PAS staining allowed for clear visualization of liver structure, bright red or red-purple glycogen particles, and blue nuclei. Optical density analysis showed higher glycogen contents in the experimental group compared with the control group (OD, 0.46 ± 0.25 vs 0.32 ± 0.18, *P* < 0.01). No differences were detected between the sham group and the control group (*P* > 0.05, Figure 1 and Table 3).

**Table 3:** Hepatic glycogen content in rats with cognitive impairment 4 weeks after removing liver sympathetic nerves \left(\bar{x}\pm s\right)

Group	*n*	Liver glycogen
Experimental group	15	0.46 ± 0.25*
Sham group	15	0.30 ± 0.22**
Control group	15	0.32 ± 0.18

Compared to the control group, *P < 0.01, **P > 0.05.

### 3.3 *Glut2* mRNA and protein expression in liver

**Figure 2:**
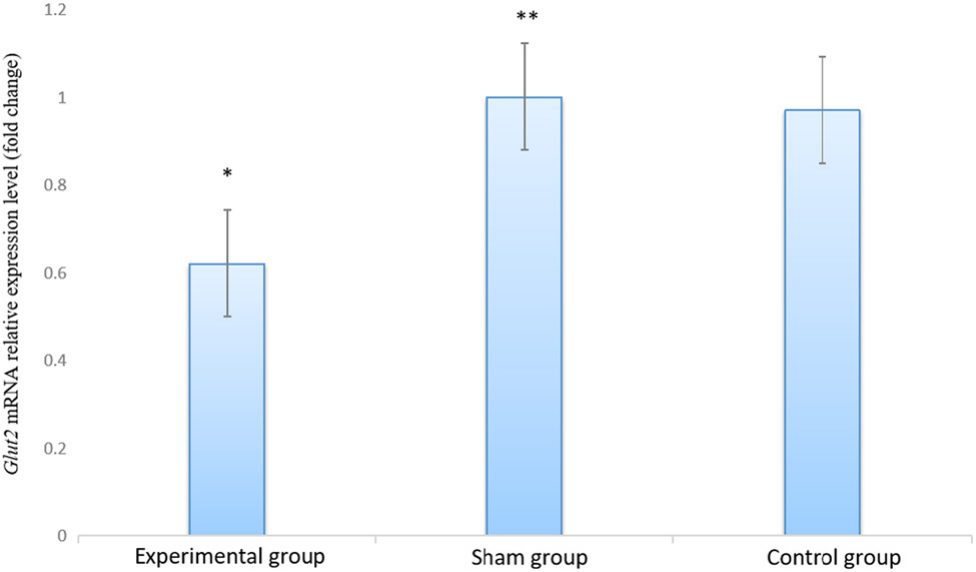
The expression level of *Glut2* mRNA in liver tissue of rats with cognitive impairment after 4 weeks of hepatic sympathetic nerve removal (compared to the control group, *P < 0.01, **P > 0.05).

**Figure 3:**
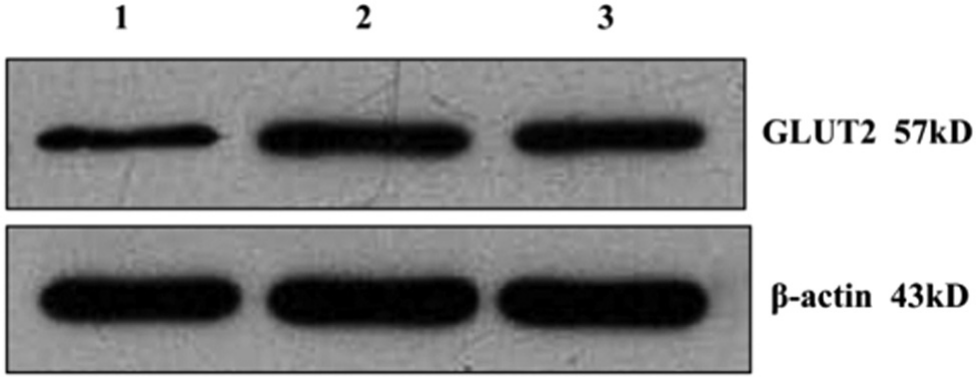
The expression of GLUT2 protein in the liver tissue of rats with cognitive impairment after 4 weeks of removal of the hepatic sympathetic nerve (1 experimental group, 2 sham group, and 3 control group).

Real-time qPCR was used to quantitate the liver *Glut2* mRNA levels at 4 weeks after 6-OHDA injection. The dissociation curve revealed a single sharp peak without nonspecific amplification products. The relative *Glut2* mRNA expression was significantly lower in the experimental group compared to the sham and control groups (0.62 ± 0.28 vs 0.97 ± 0.30, *P* < 0.01) (Figure 2). Western blot analysis showed signals at the molecular masses of GLUT2 (57 kDa) and β-actin (43 kDa). The GLUT2 protein levels were significantly lower in the experimental group than in the control group (0.39 ± 0.17 vs 0.68 ± 0.12, *P* < 0.01). No differences were detected between the sham group and the control group (*P* > 0.05, Figure 3 and Table 4).

**Table 4:** Expression of GLUT2 protein in liver tissues of rats with cognitive impairment 4 weeks after removing the liver sympathetic nerve \left(\bar{x}\pm s\right)

Group	*n*	GLUT2
Experimental group	15	0.39 ± 0.17*
Sham group	15	0.71 ± 0.20**
Control group	15	0.68 ± 0.12

Compared to the control group, *P < 0.01, **P > 0.05.

## 4 Discussion

AD and diabetes mellitus (DM) are complex diseases with overlapping clinical manifestations and pathophysiological mechanisms. Up to 81% of AD patients display abnormal glucose metabolism [14], but the underlying regulatory mechanisms remain unclear. Previous reports have suggested that cognitive function in AD declines prior to abnormal blood glucose levels, but a causal relationship has not been established [15].

Within the hippocampus and hypothalamus, the brainstem and amygdala make up the limbic system of the internal center. This region is structurally and functionally complex, as it contains a large number of afferent and efferent nerve fiber connections. Functionally, it regulates metabolic organs by controlling autonomic nerve neurotransmitter secretion and affecting the endocrine system [16,17]. The hypothalamus–pituitary–adrenal (HPA) axis is an important channel for neuroendocrine regulation. The hippocampus regulates the function of metabolic organs by inhibiting or activating the HPA axis. For example, this region stimulates the hippocampal CA3 pyramidal cells and dentate gyrus to decrease plasma cortisol release. However, damage to the ventral hippocampus structure promotes cortical hormone secretion [18,19]. Liver function, regeneration, and fibrosis are influenced by double innervation of hepatic sympathetic and parasympathetic nerves.

In hypoglycemia, increasing sympathetic nerve activity promotes liver glucose production, reduced insulin secretion, and increased glucagon secretion. In hyperglycemia, increasing parasympathetic nerve activity promotes the conversion of peripheral blood sugar into glycogen and decreases liver glucose production. In this case, the pancreas increases insulin secretion, promoting sugar utilization in muscle and adipose tissue [20]. This study involved the selective absorption of the rat hepatic sympathetic nerve by 6-OHDA treatment to establish an animal model of cognitive impairment in rats.

The results indicate that sympathetic nerve removal significantly decreased the body mass, food intake, and blood glucose levels and increased the insulin levels in treated animals. However, the TG, TC, HDL, and LDL levels were not impacted. These findings suggest that the hepatic sympathetic nerve plays an important role in hepatic metabolism. Previous studies have shown that hepatic nerves regulate glucose and lipid metabolism, food intake, and liver regeneration [21]. The visceral sensory afferent fibers of the liver portal vein transfer osmotic pressure, blood sugar, and lipid information from blood to the CNS and influence metabolism, insulin resistance, blood flow, liver steatosis, and bile secretion [22,23]. Bruinstroop et al. [24] showed that damage to the hepatic sympathetic nerve reduced the secretion of very-low-density lipoprotein-triglyceride (VLDL-TG) in obese rats and eventually led to decreased plasma TG concentrations. In contrast, removal of the parasympathetic nerve increased the TC levels in plasma. However, removal of the sympathetic nerve or parasympathetic nerve did not impact humoral factors, weight, or food intake.

GLUT2 regulates the release of glucose into the tissue fluid, senses changes in the blood glucose concentration, and regulates insulin secretion. GLUT2 functions in the liver, pancreas, small intestine, and other organs. Abnormal GLUT2 activity promotes metabolic dysfunction [25]. Our results show that the liver *Glut2* mRNA and protein levels are reduced in our cognitive impairment model, which may be related to hypoglycemia and body weight as well as the elevated glycogen content in the liver tissue after the removal of the hepatic parasympathetic nerve. This aligns with previous studies that recommend mirtazapine, a negative regulator of liver GLUT2, as an antidepressant treatment and as a method to reduce the blood glucose levels in diabetic patients [26].

In conclusion, the hepatic sympathetic nerve appears to play an important role in the regulation of metabolism in rats with cognitive impairment, and its effect on weight, blood sugar, and the liver glycogen levels may be mediated by liver GLUT2.

## References

[1] Xu J, Bartolome CL, Low CS, Yi XC, Chen CH, Wang P, et al. Genetic identification of leptin neural circuits in energy and glucose homeostases. Nature. 2018;556(7702):505–9. 10.1038/s41586-018-0049-7 PMC5920723 29670283

[2] Seoane-Collazo P, Fernø J, Gonzalez F, Diéguez C, Leis R, Nogueiras R, et al. Hypothalamic-autonomic control of energy homeostasis. Endocrine. 2015;50(2):276–91. 10.1007/s12020-015-0658-y 26089260

[3] Itoi K, Motoike I, Liu Y, Clokie S, Iwasaki Y, Uchida K, et al. Genome-wide analysis of glucocorticoid-responsive transcripts in the hypothalamic paraventricular region of male rats. Endocrinology. 2019;160(1):38–54. 10.1210/en.2018-00535 PMC6302960 30364965

[4] Bouret SG, Draper SJ, Simerly RB. Trophic action of leptin on hypothalamic neurons that regulate feeding. Science. 2004;304(5667):108–10. 10.1126/science.1095004 15064420

[5] Folgueira C, Beiroa D, Callon A, AI-Massadi O, Barja-Fernandez S, Senra A, et al. Uroguanylin action in the brain reduces weight gain in obese mice via different efferent autonomic pathways. Diabetes. 2016;65(2):421–32. 10.2337/db15-0889 26566631

[6] Nishizawa M, Nakabayashi H, Uehara K, Nakagawa A, Uchida K, Koya D. Intraportal GLP-1 stimulates insulin secretion predominantly through the hepatoportal-pancreatic vagal reflex pathways. Am J Physiol Endocrinol Metab. 2013;305(3):E376–87. 10.1152/ajpendo.00565.2012 23715725

[7] López-Soldado I, Fuentes-Romero R, Duran J, Guinovart J. Effects of hepatic glycogen on food intake and glucose homeostasis are mediated by the vagus nerve in mice. Diabetologia. 2017;60(6):1076–83. 10.1007/s00125-017-4240-4 28299379

[8] Malbert CH, Picq C, Divoux JL, Henry C, Horowitz M. Obesity-associated alterations in glucose metabolism are reversed by chronic bilateral stimulation of the abdominal vagus nerve. Diabetes. 2017;66(4):848–57. 10.2337/db16-0847 28082456

[9] Imai J, Katagiri H, Yamada T, Ishigaki T, Suzuki T, Kudo H, et al. Regulation of pancreatic beta cell mass by neuronal signals from the liver. Science. 2008;322(5905):1250–4. 10.1126/science.1163971 19023081

[10] O’Leary OF, Ogbonnaya ES, Felice D, Levone BR, Conroy LC, Fitzgerald P, et al. The vagus nerve modulates BDNF expression and neurogenesis in the hippocampus. Eur Neuropsychopharmacol. 2018;28(2):307–16. 10.1016/j.euroneuro.2017.12.004 29426666

[11] Ganmore I, Beeri MS. The chicken or the egg? Does glycaemic control predict cognitive function or the other way around? Diabetologia. 2018;61(9):1913–7. 10.1007/s00125-018-4689-9 30003308

[12] Xia C, Zhu L, Shao W, Mi S, Du S, Ye L, et al. The effect of hippocampal cognitive impairment and XIAP on glucose and lipids metabolism in rats. Cell Physiol Biochem. 2016;38(2):609–18. 10.1159/000438654 26845572

[13] Lin J, Peng Y, Wang S, Lai M, Young T, Salter DM, et al. Sympathetic nervous system control of carbon tetrachloride-induced oxidative stress in liver through-adrenergic signaling. Oxid Med Cell Longev. 2016;2016:3190617. 10.1155/2016/3190617 PMC4699022 26798417

[14] Biessels GJ, Staekenborg S, Brunner E, Brayne C, Scheltens P. Risk of dementia in diabetes mellitus: a systematic review. Lancet Neurol. 2006;5(1):64–74. 10.1016/S1474-4422(05)70284-2 16361024

[15] Altschul DM, Starr JM, Deary IJ. Cognitive function in early and later life is associated with blood glucose in older individuals: analysis of the Lothian Birth Cohort of 1936. Diabetologia. 2018;61(9):1946–55. 10.1007/s00125-018-4645-8 PMC6096629 29860628

[16] Leroy F, Park J, Asok A, Brann D, Meira T, Boyle L, et al. A circuit from hippocampal CA2 to lateral septum disinhibits social aggression. Nature. 2018;564(7735):213–8. 10.1038/s41586-018-0772-0 PMC6364572 30518859

[17] Cui S, Wang K, Wu SB, Zhu G, Cao J, Zhou Y, et al. Electroacupuncture modulates the activity of the hippocampus-nucleus tractus solitarius-vagus nerve pathway to reduce myocardial ischemic injury. Neural Regen Res. 2018;13(9):1609–18. 10.4103/1673-5374.237124 PMC6126117 30127122

[18] Mueller NK, Dolgas CM, Herman JP. Stressor-selective role of the ventral subiculum in regulation of neuroendocrine stress responses. Endocrinology. 2004;145(8):3763–8. 10.1210/en.2004-0097 15142982

[19] Viudez-Martínez A, García-Gutiérrez MS, Manzanares J. Cannabidiol regulates the expression of hypothalamus-pituitary-adrenal axis-related genes in response to acute restraint stress. J Psychopharmacol. 2018;32(12):1379–84. 10.1177/0269881118805495 30324842

[20] Stockhorst U, Steingrüber HJ, Scherbaum WA. Classically conditioned responses following repeated insulin and glucose administration in humans. Behav Brain Res. 2000;110(1–2):143–59. 10.1016/s0166-4328(99)00192-8 10802311

[21] Uno K, Katagiri H, Yamada T, Ishigaki Y, Ogihara T, Imai J, et al. Neuronal pathway from the liver modulates energy expenditure and systemic insulin sensitivity. Science. 2006;312(5780):1656–9. 10.1126/science.1126010 16778057

[22] Mizuno K, Ueno Y. Autonomic nervous system and the liver. Hepatol Res. 2017;47(2):160–5. 10.1111/hepr.12760 27272272

[23] Lorenzo-Martín LF, Menacho-Márquez M, Fabbiano S, et al. Vagal afferents contribute to sympathoexcitation-driven metabolic dysfunctions. J Endocrinol. 2019;240(3):483–96. 10.1530/JOE-18-0623 PMC6368248 30703063

[24] Bruinstroop E, Eliveld J, Foppen E, Busker S, Ackermans M, Filers E, et al. Hepatic denervation and dyslipidemia in obese Zucker (fa/fa) rats. Int J Obes. 2015;39(11):1655–8. 10.1038/ijo.2015.122 26134416

[25] Ahmed O, Pramfalk C, Pedrelli M, Olin M, Steffensen K, Eriksson M, et al. Genetic depletion of Soat2 diminishes hepatic steatosis via genes regulating de novo lipogenesis and by GLUT2 protein in female mice. Dig Liver Dis. 2019;51(7):1016–22. 10.1016/j.dld.2018.12.007 30630736

[26] Bektur E, Sahin E, Baycu C. Mirtazapine may show anti-hyperglycemic effect by decreasing GLUT2 through leptin and galanin expressions in the liver of type 1 diabetic rats. Iran J Basic Med Sci. 2019;22(6):676–82. 10.22038/ijbms.2019.34529.8190 PMC6570756 31231496

